# Community-engaged research to develop a Chicago violence research agenda and recommendations to support future community engagement

**DOI:** 10.1186/s40621-021-00335-9

**Published:** 2021-09-13

**Authors:** Alexander Ellyin, Kelli Day, Jacqueline Samuel, Tami Bartell, Dion McGill, Karen Sheehan, Rebecca Levin

**Affiliations:** 1 Ann & Robert H. Lurie Children’s Hospital of Chicago, 225 E. Chicago Avenue, Chicago, IL 60611 USA; 2 National Louis University, 122 S. Michigan Avenue, Chicago, IL 60603 USA

**Keywords:** Community-engaged, Violence, Partnership

## Abstract

**Background:**

Chicago has a history of gun violence with some neighborhoods, particularly Black and Brown communities, being disproportionately affected and Black male youth experiencing an even more disparate impact. Too often, violence prevention research is developed and carried out with little or no input from the people living in the most affected communities. The objective of the Community-Academic Collaboration to Prevent Violence in Chicago (CACPVC) was to bring together individuals from impacted communities with academic researchers and other community stakeholders to discuss violence and co-create a research agenda that addresses topics of mutual concern, and recommendations for engaging stakeholders including community members and organizations and funders in violence and violence prevention research.

**Methods:**

From 2014 to 2015, community members and organizations from seven defined regions across Chicago were recruited to participate. An organization network gathering was held in each region for researchers, funders, and community organization representatives to discuss violence prevention. Open community forums then took place in each community. Violence data by region was shared followed by facilitated group discussions that were recorded by youth scribes. Notes were thematically coded, grouped, and compiled after which a list of topics was refined by the CACPVC Work Group, allowing for investigator triangulation. A survey was disseminated to community stakeholders to prioritize the topics.

**Results:**

Seven network gatherings (127 attendees) and community forums (133 attendees) were held. Topic areas identified during the gatherings and forums included root causes/cycle of violence, racism and bias/structural violence, trajectory of violence, protective factors and nonviolence, geographic pattern change, violence prevention strategies, youth, family factors, community factors, school, police, gangs/street organizations, and media and public perceptions. Recommendations to support community engagement were grouped as role of research in reducing violence, role of community in violence research, relationships and respect, academic-community communication, financial considerations, training, practical considerations, research design, sharing results, communication about and use of data, and recommendations for funders.

**Conclusions:**

The violence research agenda will be used to inform community-engaged violence prevention research. The recommendations for community engagement provide a resource for researchers about topics to consider to meaningfully engage community members in future research.

## Background

In 2018, nearly 70,000 deaths and over 2 million injuries in the United States were due to violence (Centers for Disease Control and Prevention 2021). According to the Chicago Police Department (CPD), there were 27,417 violent crimes in the city of Chicago in 2018 (Chicago Police Department 2021). While the overall violent crime rate has been trending downward for the last decade (Chicago Health Atlas 2021), recent spikes in shootings and gun-related homicides in 2020 emphasize the ongoing and disproportionate impacts of violence. The Illinois Violent Death Reporting System (Ann and Robert H. Lurie Children’s Hospital of Chicago 2021) reported that, in 2017, Black youth were 13.7 times more likely to die as a result of gun violence compared to youth who were not Black (Ann and Robert H Lurie Children’s Hospital of Chicago 2021). Between 2016 and 2018, there were 7181 emergency department visits and in-patient hospitalizations for intentional injury of Chicagoans aged 0–19 years old. Hospitalization rates for violence-related injuries were highest in communities with low social, educational, and economic opportunities. These rates were five times higher for Black youth compared to White youth (Illinois Health and Hospital Association 2018).

Violence is a substantial threat to health and safety in Chicago and an issue of major public concern. Violence and its effects are important contributors to racial and ethnic health disparities across all age groups. When children are exposed to violence in their communities, schools and homes, the effects can last throughout a lifetime. Violence in a community can lead to fear and hopelessness, contributing to a devastating downward spiral in already marginalized neighborhoods.

Applying a public health approach to violence prevention means adopting consistent messages about the preventability of violence, promoting use of evidence-based violence prevention strategies, and fostering multi-sector collaboration, including engaging members of the communities most impacted by violence (Prevention Institute 2021). This approach can lead to the increased adoption of effective, sustainable violence prevention strategies, and build the capacity to develop innovative, reliable approaches to violence prevention.

Strengthening Chicago’s Youth (SCY), convened by Ann & Robert H. Lurie Children’s Hospital of Chicago (Lurie Children’s), is the largest comprehensive violence prevention collaborative in Chicago. SCY’s goal is to build capacity among public and private stakeholders to connect, collaborate, and mobilize around a public health approach to violence prevention.

The purpose of the Community-Academic Collaboration to Prevent Violence in Chicago (CACPVC), coordinated by SCY, was to build capacity to develop, implement, and evaluate strategies to reduce health disparities related to violence by establishing and enhancing connections among community organizations, researchers, and local funders working to prevent violence and strengthen youth, families, and communities in Chicago using community-engaged research (CEnR) practices. The aims of this project were to identify violence-related research priorities and infrastructure recommendations to facilitate and support community engagement in violence research.

## Methods

This community engagement project applied qualitative and quantitative research methods to gather community-level perspectives on approaches to violence prevention research.

### Research team and roles

The lead researchers in this study included violence prevention and community engagement experts from Lurie Children’s and Claretian Associates, a South Chicago community-based organization. Additional advisors included other community organization representatives from West Humboldt Park Development Council, Teamwork Englewood, and Youth Service Project and researchers from Northwestern University, University of Chicago, Chicago State University, and University of Illinois at Chicago as well as Polk Bros Foundation, a local foundation. Each of these individuals served on the CACPVC Advisory Board, which was assembled to guide the design and implementation of the study. This group met four times throughout the research phase, which took place from 2013 to 2017. The CACPVC Work Group, which consisted of academic and community organization researchers, coordinated and led the meetings. Community-based organization researchers were provided a stipend for their efforts.

The research team also included 25 youth scribes, who were high school juniors and seniors recruited by SCY in partnership with youth-serving organizations throughout Chicago. Youth scribes were trained in human research subjects’ protections and project logistics. The youth scribes recorded by hand the key points made during the group discussions at each community meeting. Youth scribes were active and compensated members of the research team, providing their own expert perspectives and input. For scribes under the age of 18 years, parent/guardian permission was required for participation. Because of Institutional Review Board concerns, while youth could participate in the research as scribes, their input at the meetings could not be collected to inform the final research products as it could not be ensured that the youth who attended the meetings had parental consent. In acknowledgement of the importance of their role in the community and in research, instead of disallowing youth from participating if their parents were not also present, youth were engaged from the beginning of the research project through their role as scribes.

### Research design

**Fig. 1:**
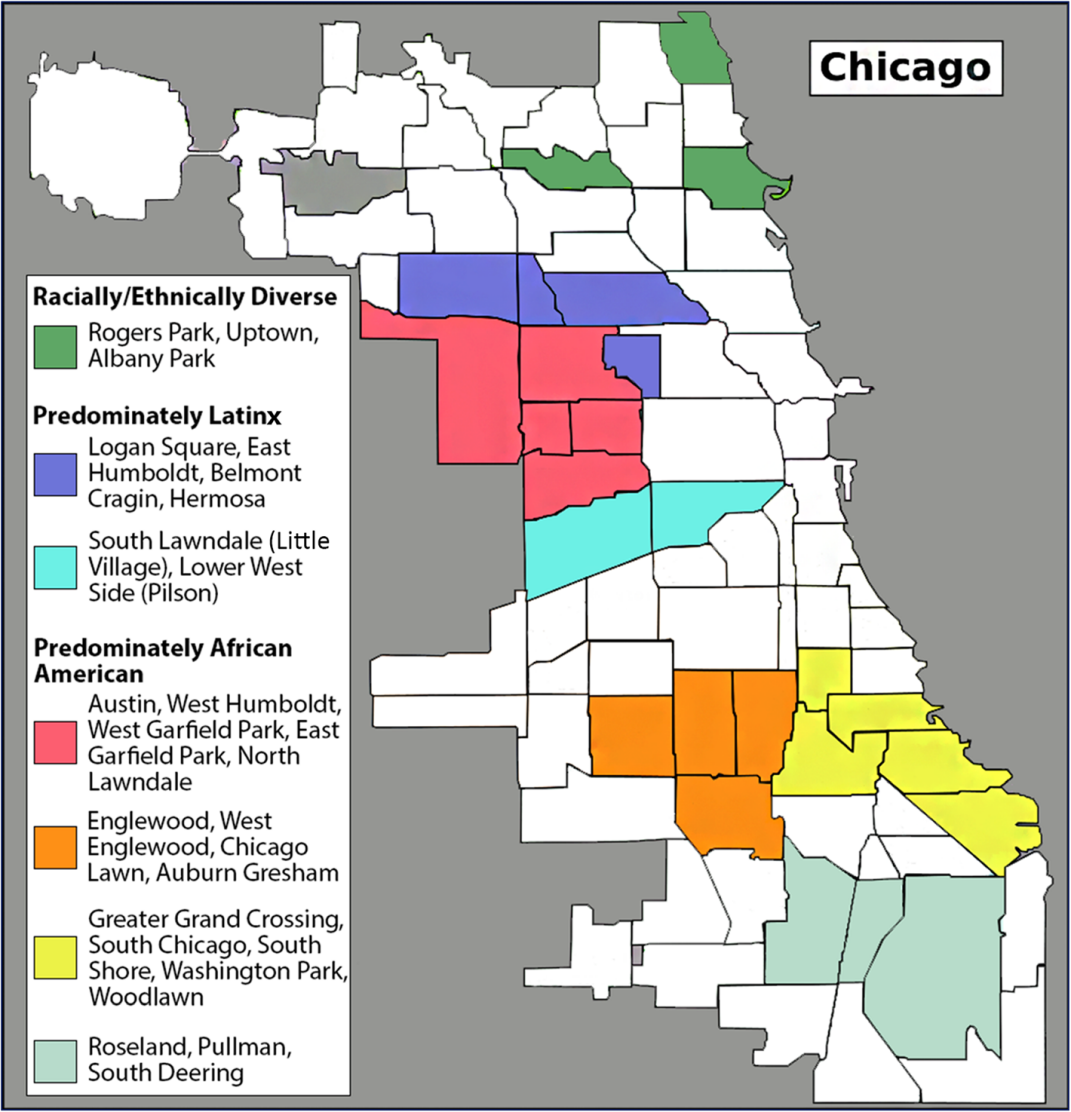
This map of Chicago depicts the communities and the racial makeup of the seven community regions that were the focus of this project

The CACPVC generated the results of this study by initially holding meetings in seven different community regions in Chicago (Fig. 1). The regions consisted of three predominantly African American communities on the South Side of Chicago, one predominantly African American community on the West Side, two predominantly Latinx communities on the West Side, and one racially, ethnically, and economically diverse community on the North Side. These community meetings were held between 2014 and 2015.

Since the information discussed at each of these meetings had potentially negative consequences for a person’s safety, health, and legal situation, several preventive steps were taken to address potential concerns. For physical risk, the Principal Investigator (RL), who has training as a Peace Circle Keeper, established rules of engagement to be followed at all meetings. To address psychological risk, each participant was given a list of curated resources. This list included information about mental health resources, such as parent classes and youth programs, and helplines, including the Domestic Violence Helpline, National Suicide Prevention Lifeline, and the Crime Victims Assistance Line. If a situation presented where a skilled mental health professional’s expertise was required, a social worker from Lurie Children’s was available to speak by telephone. The Principal Investigator informed all participants that any information shared should be kept confidential and cautioned them not to reveal any information about participation in criminal activity by themselves or others.

### Organization networking gatherings and open community forums

In each community region, an Organization Networking Gathering (ONG) and an Open Community Forum (OCF) were hosted by a community organization located in that region. Community organizations and violence prevention stakeholders were recruited to participate in the ONGs. Additional community organizations located in the convening communities were also invited to participate. Materials such as flyers, newsletter articles, emails, and Facebook posts were shared with community organizations in order to recruit community members to participate in the OCFs. Two of the OCFs were conducted in Spanish, and all materials, including flyers, infographics, resource lists, and discussion guides, were translated into Spanish for these meetings. Each meeting was open to individuals 18 years and older and each meeting lasted approximately 2 h.

All ONG/OCF meetings began with large group discussions about an infographic designed specifically for each meeting that detailed data on violence in the region where the meeting was taking place. Infographics included violent crime trends and community demographics (e.g., population race/ethnicity, employment, education, median income) compared to the city of Chicago. This was followed by a large group discussion about local violence prevention efforts. During each ONG/OCF, community organization representatives shared information about their work and participants were also informed of other violence prevention efforts in each respective region. Anywhere from one to 11 youth scribes took notes at each meeting during the large group discussions. Networking opportunities were encouraged for meeting participants.

After the large group discussions completed, participants were divided into smaller discussion groups led by a facilitator. Two youth scribes and at least one staff member took notes during these discussions, where seven key questions were asked (Table 1). The CACPVC Advisory Board and Work Group collaborated to outline the community meeting agendas and develop the small group discussion guides. These questions elicited detailed opinions on topics such as how research can impact violence, what resources stakeholders need to be able to be involved in the violence prevention effort, and what factors need to be considered so all interested individuals can participate in these efforts. After the formal discussions concluded, the youth scribes were free to participate in the discussions, though as noted, their input was not recorded for research purposes due to their age.

**Table 1:** Discussion Guide Questions

Discussion Guide Questions	1. If you were writing a newspaper story about your community, what would the headline be?
	2. Let’s talk about the data that was shared about violence in our community.
	a. What did you already know?
	i. *Prompts:* What was correct? What didn’t capture the whole picture? What was wrong?
	b. What did you learn?
	c. What do you still want to know more about?
	3. When you think about violence in Chicago and specifically in your community, are there important questions that no one is asking?
	4. What do you think the role of research is?
	*Prompt:* What value does research bring to your community? How can research help us learn more about violence? How can research help us learn how to prevent violence?
	5. Who are the important stakeholders in your community?
	6. Let’s talk about some specific roles community members can play in research. What kind of support would someone need to participate in one of these roles? What might keep someone from being able to play one of these roles?
	*Prompt:* Financial, trust, accountability, follow-up, resilience, time/convenience
	a. Being on the Advisory Board for a research project
	b. Helping to write questions for a study
	c. Collecting data, for example, surveying people in your community or using a checklist to assess the safety of your neighborhood
	d. Being a subject in a research study
	e. Helping to recruit other subjects for a research study
	f. Helping researchers make sense of their study results
	g. Sharing research results with the community
	7. How might you want to be involved in research collaboration about violence in your community?
	*Prompt:* Distinguish participation as a research subject and as a partner in conducting research.

At the conclusion of the discussion groups, all meeting participants were presented with a meeting evaluation survey. The survey asked about whether the meeting impacted their knowledge about and attitudes towards violence as well as if the meeting was satisfactory. Questions included “How much did you learn about how to prevent violence in your community at this meeting?” and “Which roles would you be willing to play for violence research in your community?” Participants who completed the survey at the OCF were entered into a raffle.

Following the meetings, the CACPVC Work Group led debriefing sessions with the youth scribes to discuss any issues or concerns and in order to ensure that the recorded notes were as accurate as possible.

### Data analysis: development of the research agenda and recommendations

The notes from each meeting were transcribed, thematically coded, grouped, and compiled. This compilation was refined by the CACPVC Work Group, thereby allowing for investigator triangulation. For the research agenda only, a survey was sent out to SCY collaborators, including community members, community organization representatives, and other violence prevention stakeholders (107 respondents), to gather further community input in identifying violence prevention research priority areas. The CACPVC Work Group collaborated with the CACPVC Advisory Board to create and refine the final violence research agenda and recommendations to support community engagement in violence research.

## Results

The results of this project included the meeting participation and evaluation responses and the development of the violence research agenda and recommendations to support community engagement in violence research.

### Meeting participation

Attendance at the ONGs ranged from 9 to 26 participants, with an average of approximately 18 participants per gathering and a total attendance of 127 participants. Attendance at the OCFs ranged from 5 to 40 participants, with an average of 19 participants per forum and a total attendance of 133 participants. Participants represented a range of community groups and professions in addition to researchers and funders, including educators, law enforcement, first responders, and others who play a role in violence prevention. While we know that there was a diverse group of individuals in attendance, we did not track participant names/affiliations because of human subject protections.

### Violence research agenda

The violence research agenda reflects the collective input of stakeholders across Chicago, including community residents and representatives of community-based organizations, academic researchers, and funders. Thirteen topic areas were compiled with each topic identifying 3 to 7 specific research questions to be addressed. Research gaps identified the need to understand how racism, structural violence, law enforcement practices, media, and where a person lives, especially in relation to segregation, all impact violence.

Specific to youth, the mutually developed agenda further seeks to understand what schools, parents, and communities can do to protect youth and prevent youth from engaging in violence.

While there are current violence prevention efforts taking place in Chicago, the research agenda also includes the need to study the effectiveness of these efforts, which includes assessing how violence prevention efforts impact violence both in the short- and long-term. Additionally, the agenda reflects the importance of violence prevention efforts being aligned with community needs.

For a complete list of violence research agenda topics and questions, see Table 2. The complete research agenda is also available on the SCY website (Strengthening Chicago’s Youth 2021).

**Table 2:** Topic areas and questions for violence research agenda

Topic Areas for Violence Research Agenda	Violence Research Agenda Questions
Root causes/cycle of violence	• What are the root causes of different types of violence in different neighborhoods? • What are the different factors that play a part in contributing to or interrupting cycles of violence? • What underlying structural factors (e.g. prison, law enforcement, school discipline) perpetuate violence? • How is sexual and gender-based violence reflected in the cycle of violence? • How is the justice system related to the cycle of violence at individual, family, community levels? • What economic drivers promote or deter violence? • What is the relationship between violence and poverty? How can the violence-poverty cycle be interrupted?
Racism and bias/structural violence	• How does racism/structural violence contribute to community violence? • What do statistical data (e.g. trends across generations, geographic differences) tell us about structural violence? • How is violence related to current and historical patterns of segregation? • How is violence related to the legacy of slavery?
Trajectory of violence	• Is there a tipping point or age of intervention when it might be necessary to transition from a prevention perspective to an intervention perspective? What are the points of intervention when people are on a trajectory toward greater involvement in violence? • What should intervention look like and when is it most effective? • What are the effects of violence and trauma on mental health? What are the effects of mental health issues on violence? • What affects the likelihood of involvement in violence for individuals returning to the community after incarceration? • For what reasons do people carry firearms?
Protective factors and nonviolence	• What are the key protective factors for youth who are not involved in violence? Why do some children grow up to be healthy and successful and others do not? • What promotes thriving in a neighborhood?
Geographic pattern change	• Why is it historically that the same communities have high rates of violence? • What does violence look like in suburbs that are demographically similar to Chicago neighborhoods with high levels of violence? • Is there something unique or specific to violence in Chicago?
Violence prevention strategies	• How do public and private funding strategies affect violence? • How effective are programs and policies at reducing and preventing violence? • How well do existing programs, policies, and funding align with the needs of youth, families, and communities? • How quickly do programs and policies have an impact on violence? How long do the effects of programs and policies last? • How do unsuccessful programs and policies affect the community?
Youth	• What do youth report as their sources of belonging and support? • What do youth report as the biggest challenges they are facing? • What do youth report they need? • What do youth report are good ways to engage and empower them? • What are the best ways for adults to support youth? • What is the role of youth in addressing violence?
Family factors	• What is the impact of the intergenerational cycle of violence? • What is the relationship between violence in the home and community violence? • What factors contribute to effective or ineffective parenting? • What are the best ways for organizations and institutions to support parenting? • How are household makeup and functioning related to violence?
Community factors	• How does violence in a community affect the overall health and well-being of the community and its residents? • How do the quality and quantity of youth-supporting services and structures affect community safety? • What is the impact of housing policy and infrastructure (e.g. tearing down housing projects, home ownership, and displacement of residents) on crime and violence? • What strategies lead to community level change in safety? • What are the characteristics of community leadership that contribute to safety? • What strategies promote authentic engagement and empowerment of community residents in ensuring safety? • How does the built environment impact community safety and well-being?
Schools	• What is the role of schools in addressing violence? • What is the impact of structure, quality, and funding of Chicago’s education system on violence? • How can the school-to-prison pipeline be interrupted? • How does school policy affect violence?
Police	• How do the Chicago Police Department’s policing practices affect the level of violence? • How does the level of violence affect the Chicago Police Department’s policing practices? • What contributes to relationships between police and community?
Gangs/street organizations	• What is the relationship between gangs/street organizations and community violence and how has it changed over time? • What is the structure of gangs/street organizations in Chicago? How has it changed over time? • Has policy (e.g., public housing reform, law enforcement strategy) affected the structure of gangs/street organizations?
Media and public perceptions	• What is the relationship between bullying/cyberbullying/social media and community violence? • What role does media play in promoting or deterring violence? • What role does media play in shaping public perception and community norms? • What role do community norms play in promoting or deterring violence? • What is the relationship between public perception of safety and what data indicate about safety? What is the impact of public perception of safety on level of violent crime in a community? • How can awareness and concern about violence be raised among those who live in communities that are less affected by violence?

### Recommendations for community engagement

The list of recommendations for researchers to support community engagement in their violence research studies detail the importance of the role of community members in research on violence, particularly gun violence and the range of actual and perceived violence that occurs in their respective communities.

The recommendations were grouped into 11 themes with 3 to 12 specific items identified for each theme. Examples of recommendations include the need for researchers to share with community members why they believe it is important to conduct violence research. Researchers should also view community members as equitable partners in projects, which requires acknowledging their expertise and being respectful of their time, by offering childcare if needed, and providing compensation. Furthermore, researchers should make sure funders understand the complexity of violence and that there is unlikely to be a short-term fix to violence. Because of this, both positive and negative evaluation findings for violence prevention efforts should be shared. Finally, the voices of current and former perpetrators of violence should be included in violence prevention research.

For a complete list of recommendations for community engagement themes, see Table 3. The complete list of all the recommendations’ themes and specific items within those themes is available on the SCY website (Strengthening Chicago’s Youth 2021).

**Table 3:** Themes for recommendations to support community engagement in violence research

Themes for Recommendations to Support Community Engagement in Violence Research	Role of research in reducing violence
	Role of community in violence research
	Relationships and respect
	Academic-community communication
	Financial considerations
	Training
	Practical considerations
	Research design
	Sharing results
	Communication about data and use of data
	Recommendations for funders

## Discussion

This 4-year effort engaged hundreds of community residents, including youth, representatives of community organizations, including local educators, law enforcement, and first responders, and academic researchers and funders to develop a violence research agenda and recommendations to support community engagement in violence and violence prevention research. This project applied community-engaged research (CEnR) practices, which involved all partners in the research process and recognized the unique strengths that each group brings, to better understand what community-engaged violence prevention research should and could look like.

The findings from the discussions used to create the research agenda highlight the variety of sectors that individuals believe play a role in or are impacted by violence, such as the education and law enforcement systems, as well as the media. This implies the valuable role that so many members of society, ranging from family members, to those who have been or are currently incarcerated, teachers, police officers, politicians, funders, and many more, can and do have in preventing violence. The recommendations to support community engagement in violence research put into words the thoughts of community members regarding their need for academic researchers to value their knowledge and expertise and take into consideration their skills, time, resources, and past experiences with academic researchers.

As the data shows that youth are victims of violence and injury, their voices need to be heard. While we engaged youth scribes in this project, we encourage other researchers to do the same and to find better ways to do so.

Since the completion of the CACPVC, SCY has partnered with Voices of Youth in Chicago Education (VOYCE) (Voices of Youth in Chicago Education 2021), a core project of Communities United, to ensure that youth are engaged in SCY’s efforts. With recent funding from the Robert Wood Johnson Foundation, Communities United, a community-based organization focusing on furthering economic, racial, and social justice efforts (Communities United 2021), has partnered with Lurie Children’s to engage 25 Boys and Young Men of Color (BYMOC) to lead a participatory action research project focused on access to mental health supports for their peers, culminating in a citywide summit and report on the issue to catalyze policy change.

The research agenda findings have further motivated our Lurie Children’s research team to perform several additional studies. For example, one study explored geographic pattern changes in violence over time. Using data from the Illinois Violent Death Reporting System, we showed the spatial and racial/ethnic changes across the city for 15- to 19-year-olds, noting how violent death “hot spots” shifted from the South Side to the West Side between 2013 and 2017 (Chadha et al. 2020). To explore one potential approach to violence prevention strategies, the Lurie Children’s team also partnered with a pediatric practice at a Federal Qualified Health Center in a community on the West Side of Chicago with high rates of community gun violence to measure attitudes toward physician counseling about firearms (Haser et al. 2020). In addition to informing research questions, the research agenda was used to direct content included in SCY’s training opportunities, which serve to support community partner’s use of data and engagement in violence research. Examples of topics have included program evaluation, human research protection, and grant writing.

This community-engaged violence research agenda is the first for Chicago. Other examples of violence prevention research agendas in the literature vary in scope. For example, there is a published research agenda set for an entire nation, for violence against women, and for pediatric firearm injuries (Ward et al. 2012; Koss 1990; Cunningham et al. 2019). The South African and the pediatric firearm research agendas include parallels to the CACPVC agenda in describing the importance of understanding how schools and community organizations can prevent violence (Ward et al. 2012; Cunningham et al. 2019). The violence against women research agenda has similarities to the CACPVC agenda with respect to understanding and analyzing intergenerational violence (Koss 1990). We will wait to see what impact this work has on the prevention of violence in each of these communities.

Although this project showed how community organizations and academics can engage with one another to set research priorities, it is not known how applicable this research agenda would be to other areas in Chicago or other cities that were not included in this project. The goal of this project was to generate Chicago-specific research priorities for communities most impacted by violence, and this research further emphasizes that these priorities should reflect and be specific to the communities for which they are developed. Therefore, the results of the CACPVC may not be generalizable to communities not included in this research. Another limitation of this project is that the last of the CACPVC community gatherings and forums took place in 2015. Therefore, there may be necessary additions or subtractions to the research agenda and the list of recommendations to support community engagement, as new developments and events occur in these communities, in Chicago, and across the country, as are happening today.

## Conclusions

This project utilized a community-engaged research (CEnR) approach to bring academic researchers, community organization leaders, and community members and youth together to discuss violence and the need for more focused and engaged violence and violence prevention research. The CACPVC specifically sought to understand how community members think that violence research should take place in their communities, as well as what violence prevention research questions should be investigated, specifically in the communities of Chicago that were represented in this project, which were those that have been most impacted by violence, including gun violence. The voices of those who are the perpetrators, victims, and witnesses of violence are essential to violence prevention research and violence prevention efforts overall.

This research agenda can inform future violence prevention projects and inform funders about what projects community organizations and community members deem important to be further explored. Additionally, the CACPVC listened to community members and collected a list of their recommendations regarding how future research should engage them and community organizations in violence prevention efforts. Future research is needed to assess how these tools have been applied by researchers and how community members have been integrated into the research process, and to evaluate if and how these combined efforts have resulted in violence prevention. Everyone has a role to play in violence prevention.

## Data Availability

The data will be made available on reasonable request to the corresponding author and conditional on Institutional Review Board approval.

## Abbreviations

BYMOC: Boys and Young Men of Color
CACPVC: Community-Academic Collaboration to Prevent Violence in Chicago
CEnR: Community-engaged research
CPD: Chicago Police Department
OCF: Open Community Forum
ONG: Organization Network Gathering
SCY: Strengthening Chicago’s Youth
VOYCE: Voices of Youth in Chicago Education
